# A novel LAMB2 gene mutation associated with a severe phenotype in a neonate with Pierson syndrome

**DOI:** 10.1186/s40001-016-0215-z

**Published:** 2016-04-30

**Authors:** Boutaina Zemrani, François Cachat, Olivier Bonny, Eric Giannoni, Jacques Durig, Florence Fellmann, Hassib Chehade

**Affiliations:** Division of Pediatric Nephrology, Department of Pediatrics, Lausanne University Hospital, Rue du Bugnon 46, 1011 Lausanne, Switzerland; Service of Nephrology, Lausanne University Hospital, Rue du Bugnon 21, 1011 Lausanne, Switzerland; Service of Neonatology, Lausanne University Hospital, Rue du Bugnon 46, 1011 Lausanne, Switzerland; Service of Ophthalmology, Lausanne University Hospital, Rue du Bugnon 46, 1011 Lausanne, Switzerland; Service of Medical Genetics, Lausanne University Hospital, Rue du Bugnon 46, 1011 Lausanne, Switzerland

**Keywords:** Pierson syndrome, Newborn, Nephrotic syndrome, LAMB2 mutation

## Abstract

**Background:**

Pierson syndrome (PS) is a rare autosomal recessive disorder, caused by mutations in the laminin β2 (LAMB2) gene. It is characterized by congenital nephrotic syndrome, microcoria, and neurodevelopmental deficits. Several mutations with genotype–phenotype correlations have been reported, often with great clinical variability. We hereby report a novel homozygous nonsense mutation in the LAMB2 gene, associated with a severe phenotype presentation.

**Case diagnosis:**

We describe a term male infant born from consanguineous parents. The mother previously lost three children in the neonatal period, secondary to undefined renal disease, had two spontaneous abortions, and gave birth to one healthy daughter. The index case presented at birth with bilateral microcoria, severe hypotonia, respiratory distress, and congenital nephrotic syndrome associated with anuria and severe renal failure requiring peritoneal dialysis. The patients’ clinical follow-up was unfavorable, and the newborn died at 7 days of life, after withdrawal of life support. Genetic analysis revealed a homozygous nonsense mutation at position c.2890C>T causing a premature stop codon (p.R964*) in LAMB2 gene.

**Conclusion:**

We here describe a novel nonsense homozygous mutation in LAMB2 gene causing a severe neonatal presentation of Pierson syndrome. This new mutation expands the genotype–phenotype spectrum of this rare disease and confirms that truncating mutations might be associated with severe clinical features.

## Background

Pierson syndrome (PS) is a rare autosomal recessive disease, characterized by congenital nephrotic syndrome and progressive renal failure [1]. It can be also associated with ocular abnormalities and neurodevelopmental deficits [1]. The incidence of PS remains unknown, although PS is recognized as the fourth most common cause of congenital nephrotic syndrome [2]. Affected individuals have mutations in the LAMB2 gene, coding for one of several extracellular glycoproteins expressed in the glomerular basement membrane, ocular structures, and the neuromuscular synapses [1].

We here report a novel homozygous nonsense mutation at position c.2890C>T in the LAMB2 gene causing a premature stop codon (p.R964*) in a newborn, associated with a severe phenotype presentation.

## Case presentation

**Fig. 1:**
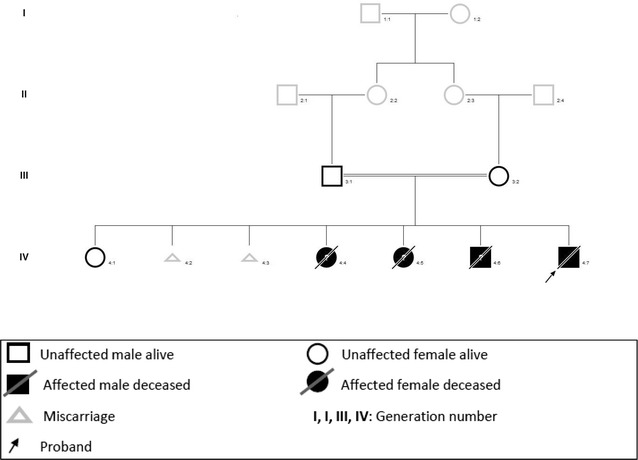
Genealogical tree of the family: Parents are first-cousins; three sibs of the proband died in the neonatal period and were probably affected by Pierson’s syndrome

**Fig. 2:**
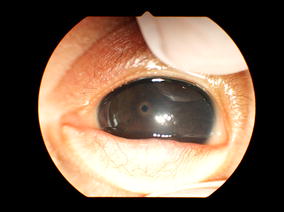
Narrow nonreactive pupils (microcoria)

The mother of the index patient was 25 years old at the time of birth. Parents were consanguineous cousins. She previously had six pregnancies, but lost all of them except one (a healthy girl). Three of her previous infants died in Somalia from undefined renal disease (two girls in the immediate neonatal period, and one boy at 1 month of age). The mother also had two spontaneous abortions (Fig. 1). Current pregnancy was complicated by bilateral hydronephrosis in the fetus, diagnosed at 22 weeks of gestation. Prenatal ultrasounds revealed hyperechogenic kidneys, associated with oligohydramnios at 32 weeks of gestation, and anamnios at 34 weeks of gestation requiring a C-section. At birth, the index patient presented with severe respiratory distress and persistent pulmonary hypertension requiring mechanical ventilation. Physical exam was remarkable for severe hypotonia and bilateral microcoria (Fig. 2). Birth weight, length, and head circumference were 2380 g (P10-50), 46.5 cm (P10-50), and 31 cm (P10-50), respectively. Placental weight was 840 g (>P97). Laboratory investigations revealed a severe nephrotic range proteinuria with acute renal failure [total plasma protein 30 g/l (reference range 46–70 g/l), albumin 16 g/l (reference range 26–36 g/l), urine protein 58.5 g/l (reference range ≤0.15 g/l), and plasma creatinine 300 μmol/l (reference range 17.7–88.4 umol/l)]. Peritoneal dialysis was initiated with daily albumin infusions. Renal ultrasonography showed enlarged hyperechogenic kidneys with a poor corticomedullary differentiation.

On the basis of the physical examination and clinical presentation, Pierson syndrome was suspected. Genetic analysis was performed in the Blueprint Genetics laboratory in Finland, using “the Nephrotic syndrome panel” which covers nine genes with a strong association with this disorder. Pierson syndrome was confirmed, with a novel homozygous nonsense mutation c.2890C>T (p.R964*) in the LAMB2 gene. Due to the dismal prognosis, life support treatment was withdrawn, and the infant died at 7 days of life.

## Discussion

PS was first described in 1963 by Michel Pierson [1, 3, 4]. It is caused by mutations in the LAMB2 gene. It’s a rare disease with only around 50 mutations from about 40 unrelated families identified so far [1]. Recently, several novel mutations in LAMB2 gene that expand the clinical spectrum of LAMB2-associated disorders have been described [1, 2, 5–8]. Disease-causing mutations comprise missense, nonsense, and splice-site mutations, as well as small deletions and insertions, found either as homozygous or compound heterozygous. Truncating mutations are associated with severe disease. However, nontruncating LAMB2 mutations result in partial expression of laminin β2, causing less severe phenotype, resulting in some residual function of the gene [9, 10] and milder or atypical phenotypes [1, 2, 8, 9].

Kidney involvement in PS seems to be a universal presentation, with variable degrees of severity [8], ranging from a minor podocyte effacement to a diffuse mesangial sclerosis with crescentic formation [2]. Of note, renal involvement can be present before birth, as in our case, or appear later in life.

Ocular involvement is also an important finding in PS, the most ocular anomaly being microcoria. Other ocular abnormalities have been described, such as iris anomalies, abnormal lens shape with cataracts, retinal abnormalities or high-grade myopia [4, 6, 11, 12]. Ocular anomalies are not universal in PS patients. They can even be lacking [11], as it seems that only little residual function of LAMB2 is required for normal development of iris muscles [2].

Many patients with PS show severe neurodevelopmental deficits and muscular weakness [13]; however, similar to the eye involvement, patients with truncating or splice-site mutations on each allele can present normal neurologic and cognitive development [6, 8, 13].

Current data suggest the presence of significant genotype–phenotype correlations [4, 12]. In fact, some authors have shown that patients with mild phenotypes (including minor glomerular changes or focal and segmental glomerulosclerosis) harbor at least one missense or nontruncating deletion mutation, whereas patients with more severe phenotypes (including mesangial sclerosis, severe renal symptoms or end-stage renal disease in the first year of life) have truncating mutations [1, 4, 8, 12, 14]. However, recent reports show that patients with truncating mutations do not always harbor such severe features: two Asian subjects with bi-allelic truncated mutations kept a normal renal function until 3 and 6 years of age [6, 15]. Our patient has a nonsense mutation, resulting in a premature truncation of laminin β2 (stop codon after 964 residues out of 1798). It caused a severe phenotype, leading to end-stage renal failure in the early neonatal period. This mutation is reported for the first time in a homozygous state. According to EXAC database (http://www.exac.broadinstitute.org/August 2015), there’s one similar mutation (R964*) in a heterozygote pattern found using a genomic sequencing in 60,000 individuals. The prevalence of heterozygous truncating mutations in the EXAC database is about 1/1000 person, leading to a probability of 1 over 4 million to have a PS with a truncating mutation. PS has rarely been described in African subjects [1]. Although ethnicity might play a role, this is most likely secondary to underdiagnoses of PS in Africa.

## Conclusion

Clinical spectrum of LAMB2-associated disorders varies from mild-to-severe ocular, renal, and neurologic involvement. This case adds to the literature a severe and fatal neonatal form of PS secondary to a newly nonsense homozygous mutation in the LAMB2 gene. This new mutation expands the genotype–phenotype spectrum of this rare disease and confirms that truncating mutations might be associated with a severe clinical phenotype.

## Consent

Written informed consent was obtained from the patient for publication of this case report and any accompanying images.
